# Genome‐wide SNPs resolve spatiotemporal patterns of connectivity within striped marlin (*Kajikia audax*), a broadly distributed and highly migratory pelagic species

**DOI:** 10.1111/eva.12892

**Published:** 2019-11-22

**Authors:** Nadya R. Mamoozadeh, John E. Graves, Jan R. McDowell

**Affiliations:** ^1^ Department of Fisheries Science Virginia Institute of Marine Science William & Mary Gloucester Point Virginia

**Keywords:** DArT, genetic connectivity, highly migratory, large pelagic fish, population genomics, SNPs, striped marlin, temporal stability

## Abstract

Genomic methodologies offer unprecedented opportunities for statistically robust studies of species broadly distributed in environments conducive to high gene flow, providing valuable information for wildlife conservation and management. Here, we sequence restriction site‐associated DNA to characterize genome‐wide single nucleotide polymorphisms (SNPs) in a broadly distributed and highly migratory large pelagic fish, striped marlin (*Kajikia audax*). Assessment of over 4,000 SNPs resolved spatiotemporal patterns of genetic connectivity throughout the species range in the Pacific and, for the first time, Indian oceans. Individual‐based cluster analyses identified six genetically distinct populations corresponding with the western Indian, eastern Indian, western South Pacific, and eastern central Pacific oceans, as well as two populations in the North Pacific Ocean (*F*_ST_ = 0.0137–0.0819). *F*_ST_ outlier analyses identified a subset of SNPs (*n* = 59) putatively under the influence of natural selection and capable of resolving populations separated by comparatively high degrees of genetic differentiation. Temporal collections available for some regions demonstrated the stability of allele frequencies over three to five generations of striped marlin. Relative migration rates reflected lower levels of genetic connectivity between Indian Ocean populations (*m*_R_ ≤ 0.37) compared with most populations in the Pacific Ocean (*m*_R_ ≥ 0.57) and highlight the importance of the western South Pacific in facilitating gene flow between ocean basins. Collectively, our results provide novel insights into rangewide population structure for striped marlin and highlight substantial inconsistencies between genetically distinct populations and stocks currently recognized for fisheries management. More broadly, we demonstrate that species capable of long‐distance dispersal in environments lacking obvious physical barriers to movement can display substantial population subdivision that persists over multiple generations and that may be facilitated by both neutral and adaptive processes. Importantly, surveys of genome‐wide markers enable inference of population‐level relationships using sample sizes practical for large pelagic fishes of conservation concern.

## 1 INTRODUCTION

Genetic studies of wild populations have assisted the development of scientifically informed management and conservation efforts over the past five decades (Frankel, 1974; Franklin, 1980; Simberloff, 1988; Soulé, 1985). However, for many species of conservation concern, information on spatiotemporal patterns of genetic connectivity remains limited, challenging the ability of resource managers to develop recovery plans that consider genetic attributes of distinct populations (Frankham, 2010; Funk, McKay, Hohenlohe, & Allendorf, 2012; Palsbøll, Bérubé, & Allendorf, 2006). This is frequently the case for large pelagic fishes, which display broad spatial distributions and highly migratory life histories, and seasonally occupy multiple domestic and international management jurisdictions (Meltzer, 1994). The advent of next‐generation sequencing (NGS; e.g., Mardis, 2008) represents a particularly important advancement in genetic insights possible with sampling designs practicable for large pelagic fishes, especially those that also occur in low frequency. Surveys of genome‐wide variation are capable of providing new information on population structure and movement patterns to improve conservation and management efforts for these species.

The now widespread availability of NGS enables rapid and cost‐effective surveys of thousands to hundreds of thousands of molecular markers across entire genomes, facilitating statistically robust assessments of neutral and adaptive genomic variation in nonmodel systems. Though such assessments have progressed toward unraveling complex relationships between fitness‐related traits and underlying genomic architectures in more easily accessible systems (e.g., Pacific salmonids [*Oncorhynchus* spp., *Salmo* spp.]; Ayllon et al., 2015; Barson et al., 2015; Prince et al., 2017; Thompson et al., 2019), applications of NGS to large pelagic fishes are just getting started, and recent studies illustrate the utility of genomic methods for resolving spatial patterns of connectivity in pelagic systems. For example, results from early comparisons of traditional markers (e.g., allozymes, mtDNA, microsatellites) in yellowfin tuna (*Thunnus albacares*) from the Atlantic, Pacific, and Indian oceans were either consistent with genetic homogeneity or offered only preliminary evidence for population subdivision (Appleyard, Grewe, Innes, & Ward, 2001; Dammannagoda, Hurwood, & Mather, 2008; Díaz‐Jaimes & Uribe‐Alcocer, 2003, 2006; Ely et al., 2005; Scoles & Graves, 1993; Ward, Eiliott, Grewe, Smolenski, & Sea, 1994; Wu et al., 2010). In comparison, recent surveys of genome‐wide single nucleotide polymorphisms (SNPs) provide definitive evidence for genetically distinct populations of yellowfin tuna within and between ocean basins (Barth, Damerau, Matschiner, Jentoft, & Hanel, 2017; Grewe et al., 2015; Mullins, McKeown, Sauer, & Shaw, 2018; Pecoraro et al., 2016), supplying important information to improve management efforts for this commercially harvested species. Applications of NGS to large pelagic fishes have so far prioritized species of high commercial value that also occur in large numbers (e.g., schooling species). Considerable potential exists for genomic surveys to provide valuable information for additional large pelagic fishes of conservation concern, including those found at comparatively lower frequencies or of lesser commercial value.

Striped marlin (*Kajikia audax*) is a large pelagic fish broadly distributed in temperate, subtropical, and tropical waters of the Pacific and Indian oceans (Nakamura, 1985), and supports valuable recreational and commercial fisheries throughout the species range. Tagging studies demonstrate that striped marlin is capable of long‐distance movements spanning hundreds to thousands of kilometers over periods less than one year (Domeier, 2006; Holdsworth, Sippel, & Block, 2009; Ortiz et al., 2003; Sippel, Davie, Holdsworth, & Block, 2007), presumably to exploit seasonal spawning and feeding grounds. Despite such high dispersal capabilities, striped marlin display some degree of site fidelity to regions largely corresponding with known spawning grounds (Domeier, 2006; Ortiz et al., 2003). Genetic studies based on microsatellites and mtDNA provide evidence for at least four genetically distinct populations of striped marlin in the Pacific Ocean (McDowell & Graves, 2008; Purcell & Edmands, 2011), and these results are generally consistent with available information on seasonal movements and spawning behavior. However, incongruities in results between genetic studies reflect uncertain population‐level relationships for striped marlin in the central North Pacific and eastern central Pacific oceans, complicating the delineation of biologically relevant management units in these regions. Additionally, population structure for striped marlin in the Indian Ocean remains unexplored, and the degree of genetic connectivity between ocean basins is unknown.

Practical challenges to implementing biologically representative sampling designs have impeded rangewide studies of biological and genetic relationships in striped marlin. Catches of striped marlin outside of seasonal feeding or spawning assemblages are typically low, and known assemblages are often difficult to access. The location and timing of striped marlin spawning are also poorly understood, and few efforts exist to sample larvae or reproductively active adults. These challenges have resulted in a lack of information on spatial genetic variation across the full range of striped marlin, leading to mismatches between populations characterized by distinct biological processes and stocks recognized by regional fisheries management organizations (RFMOs). For example, a single ocean‐wide stock is presently recognized in RFMO assessment and management efforts in the Indian Ocean due to insufficient information on population structure. Additionally, uncertain population structure in some regions of the Pacific Ocean is further complicated by continued use of stock boundaries inconsistent with populations evident from available genetic and biological information. Such mismatches may be especially problematic for striped marlin because this species is estimated to be overfished or experiencing unsustainable levels of fishing effort in regions across the Indo‐Pacific (IATTC, 2018; WCPFC, 2012, 2018). Studies that provide information to improve rangewide management and conservation efforts for striped marlin are timely not only because of the unsustainable status of most stocks (Collette et al., 2011; Cullis‐Suzuki & Pauly, 2010), but also because habitat utilization and seasonal movements of striped marlin and other large pelagic fishes rely on environmental cues progressively influenced by a changing global climate (Carlisle et al., 2017; Dell'Apa, Carney, Davenport, & Vernon, 2018; Duery, Bopp, & Maury, 2014; Hazen et al., 2012; Mislan, Deutsch, Brill, Dunne, & Sarmiento, 2017; Muhling, Lee, Lamkin, & Liu, 2011; Pentz, Klenk, Ogle, & Fisher, 2018).

Here, we employ NGS of restriction site‐associated DNA (Andrews, Good, Miller, Luikart, & Hohenlohe, 2016; Baird et al., 2008) to assess spatiotemporal patterns of genetic variation in striped marlin across the Pacific and Indian oceans. Relative to previous genetic studies of striped marlin, NGS‐based methodology facilitates statistically robust assessment of genome‐wide variation (Helyar et al., 2011; Luikart, England, Tallmon, Jordan, & Taberlet, 2003; Nielsen, Hemmer‐Hansen, Larsen, & Bekkevold, 2009) despite pragmatic constraints on sampling efforts for this species. The primary objectives of this study were to: (a) determine the number and geographic extent of striped marlin populations in the Pacific and Indian oceans, (b) evaluate whether genetically distinct populations correspond with genetic variation potentially influenced by natural selection, and (c) assess the multigenerational stability of observed genomic variation. This work represents the first genomic assessment of striped marlin, and results provide novel insights into genetic connectivity within and between ocean basins, the role of putatively neutral and adaptive processes in facilitating population subdivision, and the stability of allele frequencies over decadal time periods.

## 2 METHODS

### 2.1 Sample collection and DNA isolation

**Figure 1:**
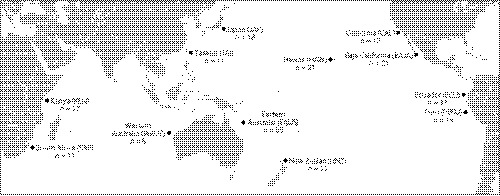
Map displaying sampling locations and sample sizes for striped marlin (*Kajikia audax*) evaluated in this study. Points correspond with representative sampling region

Striped marlin tissue samples were opportunistically collected during the period 1992 through 2017 from locations across the species range, including waters off South Africa (SAF), Kenya (KEN), and northwestern Australia (WAUS) in the Indian Ocean, and eastern Australia (EAUS), New Zealand (NZ), Japan (JAP), Taiwan (TAI), Hawaii (HAW), southern California (CAL), Baja California (BAJA), Ecuador (ECU), and Peru (PERU) in the Pacific Ocean (Table 1; Figure 1). Samples consisted of fin tissue from striped marlin released alive following capture by recreational anglers or from striped marlin caught incidentally by commercial pelagic longline vessels targeting tunas and swordfish. Additional samples consisting of muscle tissue were obtained from local markets. All samples were preserved in 95% ethanol or a 10% dimethyl sulfoxide solution (Seutin, White, & Boag, 1991) and maintained at room temperature until DNA isolation. Total genomic DNA was isolated from tissues using a DNeasy Blood & Tissue Kit (Qiagen) or a ZR‐96 Genomic DNA Kit (Zymo Research). DNA from each sample was visualized on 1.5% agarose gels, and isolations that recovered high molecular weight DNA were quantified using a Qubit 2 fluorometer and dsDNA BR assays (Thermo Fisher Scientific). Isolations with sufficient DNA for NGS were normalized to 700 ng total DNA at 50 ng per µL and stabilized in GenTegra‐DNA (GenTegra LLC). Stabilized high‐quality DNA was submitted to Diversity Arrays Technology Pty. Ltd. (DArT PL; Canberra, Australia) for DArTseq™ 1.0 genotyping.

**Table 1:** Details for striped marlin (*Kajikia audax*) samples analyzed in this study

Sampling region	Code	Year	No. of individuals	Total
Indian Ocean				
South Africa	SAF	2017	1	11
		2016	3	
		2015	7	
Kenya	KEN	2016	13	27
		2015	14	
Western Australia	WAUS	2016	8	8
			Total	46
Pacific Ocean				
Eastern Australia	EAUS	2015	3	35
		2012	3	
		2011	7	
		2010	6	
		1994	16	
New Zealand	NZ	2017	22	22
Japan	JAP	2015	18	18
Taiwan	TAI	2016	4	11
		2015	5	
		2014	2	
Hawaii	HAW	2015	21	21
California	CAL	2016	2	15
		2000	13	
Baja California	BAJA	2015	21	21
Ecuador	ECU	2016	22	37
		1992	15	
Peru	PERU	2016	19	19
			Total	199
		Grand total		245

### 2.2 DArTseq™ 1.0 genotyping

DArTseq™ genotyping (Sansaloni et al., 2011) involves genomic complexity reduction followed by NGS and is similar to other commonly utilized approaches for NGS of reduced genomic representations (e.g., double‐digest restriction site‐associated DNA sequencing; Peterson, Weber, Kay, Fisher, & Hoekstra, 2012). Genomic complexity reduction was principally performed as described in Kilian et al. (2012), but with a double restriction enzyme (RE) digestion and ligation with RE‐specific adapters. Four RE combinations were tested at the DArT PL facility, and digestion with *Pst*I and *Sph*I was selected based on the size of the representation and the fraction of the genome selected. Custom proprietary adapters used in ligation reactions were similar to those described by Elshire et al. (2011) and Kilian et al. (2012) (see Appendix S1 for detailed information). Samples were normalized and pooled at equimolar ratios into multiplex libraries each comprising 94 samples and two controls, and sequenced for 77 cycles of single‐end sequencing on single lanes of an Illumina HiSeq 2500 platform (Illumina, Inc.). Resulting sequence data were analyzed in a proprietary DArTseq™ analytical software pipeline, wherein demultiplexing, quality filtering, variant calling, and generation of final genotypes were performed in sequential primary and secondary workflows (see Appendix S1). DArT PL supplied a final genotype matrix containing 61,908 SNP loci and metadata associated with each locus.

### 2.3 SNP quality filtering

Additional quality filtering of SNP data received from DArT PL was performed in R version 3.3.1 (R Core Team, 2017) using the *dartR* v0.93 package (Gruber, Unmack, Berry, & Georges, 2018). Loci missing ≥10% of genotype calls were excluded from the dataset. Samples missing ≥20% of genotype calls were also excluded. To retain only high‐quality SNPs with reliable genotype calls, loci with average reproducibility <95% were removed. All monomorphic loci were also removed. In instances where more than one SNP originated from a read alignment, a single SNP was randomly retained to reduce the probability of linked loci in the final dataset. Finally, any locus with a minor allele frequency <0.05 across all samples was removed to reduce the probability of PCR error or ascertainment bias resulting from nonrandom sampling of a gene pool (Bradbury et al., 2011; Roesti, Salzburger, & Berner, 2012).

### 2.4 Statistical analyses

#### 2.4.1 Identification of genetically distinct populations

We employed multivariate analyses and Bayesian‐based simulations to infer the number and geographic extent of genetically distinct populations of striped marlin represented in our dataset. Multivariate methods were selected for exploring population structure because these methods are computationally efficient and unconstrained by assumptions of Hardy–Weinberg equilibrium (HWE; Jombart, Pontier, & Dufour, 2009). Principal coordinate analysis (PCoA) and discriminant analysis of principal components (DAPC; Jombart, Devillard, & Balloux, 2010) were performed using the R packages *dartR* and *adegenet* v2.0.1 (Jombart, 2008), respectively. Individuals were assigned to groups prior to DAPC using successive *K*‐means clustering. The most likely values for *K* were determined by generating a Bayesian information criterion (BIC) score for each *K* scenario and selecting those scenarios with the lowest BIC scores to assess with DAPC.

Population structure was also evaluated using the Bayesian simulation algorithm implemented in STRUCTURE v2.3.4 (Falush, Stephens, & Pritchard, 2003; Hubisz, Falush, Stephens, & Pritchard, 2009; Pritchard, Stephens, & Donnelly, 2000). Because STRUCTURE assumes loci conform to the expectations of HWE, we first identified loci that violated these expectations using the exact methodological approach described by Wigginton, Cutler, and Abecasis (2005). HWE was evaluated within sample collections organized by sampling location, and statistical significance of HWE comparisons was determined using a critical value corrected by a modified false discovery rate (Benjamini & Yekutieli, 2001; Narum, 2006). Loci that did not conform to the expectations of HWE in more than one sample collection were removed. All STRUCTURE analyses were performed using an admixture model of ancestry (Falush et al., 2003), a burn‐in of 50,000 followed by 500,000 Markov chain Monte Carlo simulations, and three iterations of each *K*. Default values were used for all other STRUCTURE settings, including the lack of a location prior. Previous evaluations of STRUCTURE performance demonstrate that the presence of strongly differentiated genetic clusters may obfuscate resolution of weakly differentiated clusters (Janes et al., 2017; Vähä & Primmer, 2006; Waples & Gaggiotti, 2006). In preliminary analyses of our data, the highest levels of genetic differentiation were observed between striped marlin sampled from the western Indian Ocean and the northern and eastern Pacific Ocean. Thus, to improve resolution of more weakly differentiated clusters, we performed STRUCTURE analyses on three datasets: (a) all sample collections, (b) all Pacific Ocean and eastern Indian Ocean sample collections, and (c) all Indian Ocean and western South Pacific Ocean sample collections. Scenarios with *K* equal to two through eight were evaluated for each dataset. Results for each dataset were summarized in CLUMPP v1.1.2 (Jakobsson & Rosenberg, 2007) and visualized in DISTRUCT v1.1 (Rosenberg, 2004). The most likely *K* for each dataset was identified using Structure Harvester v0.6.94 (Earl & vonHoldt, 2012; Evanno, Regnaut, & Goudet, 2005). Results from multivariate analyses and STRUCTURE simulations were collectively evaluated to determine the most likely scenario of spatial population structure for the striped marlin represented in our dataset. Based on this information, sample collections were combined into groups representing genetically distinct populations.

#### 2.4.2 SNPs putatively influenced by natural selection

To reduce the probability of committing type I or type II statistical errors (Lotterhos & Whitlock, 2014; Narum & Hess, 2011), we employed two approaches for identifying SNPs putatively under the influence of natural selection using a dataset in which loci not conforming to the expectations of HWE were removed. BayeScan v2.1 (Foll & Gaggiotti, 2008) implements a Bayesian‐based algorithm that compares allele frequencies among populations to directly estimate the probability that each locus is exposed to natural selection (Beaumont & Balding, 2004; Foll & Gaggiotti, 2008). We performed BayeScan analyses using 10,000 iterations each for the burn‐in, pilot runs, and final runs. We also used conservative prior odds for the neutral model (100:1) to reduce the probability of false positives in BayeScan results (Lotterhos & Whitlock, 2014). *F*_ST_ outlier loci were identified from BayeScan output using a false discovery rate of 0.10. Loci putatively under the influence of natural selection were also identified using the FDIST2 outlier detection method (Beaumont & Nichols, 1996; Excoffier, Hofer, & Foll, 2009) implemented in Arlequin v3.5 (Excoffier & Lischer, 2010). This method assumes a finite island model of migration to obtain a distribution of *F*_ST_ values across loci as a function of average heterozygosity within populations. Arlequin outlier detection analyses were performed with 500,000 simulations, and statistical significance was assessed using a *p*‐value of .05. A final list of SNPs putatively under the influence of natural selection included only those loci identified as outliers in both analyses. Because accurately distinguishing between loci exhibiting low levels of divergence due to balancing selection rather than neutral processes is challenging (Lotterhos & Whitlock, 2014; Whitlock & Lotterhos, 2015), particularly in high gene flow species, SNPs identified as putatively experiencing balancing selection were excluded from the final list of outlier loci. Sequences corresponding with final outlier loci were queried in an NCBI GenBank BLASTn v2.8.1 (Zhang, Schwartz, Wagner, & Miller, 2000) search to identify putative functions (search performed 17 March 2019). We only considered BLASTn hits with expect values <10^−10^.

To assess the relative contribution of SNPs putatively influenced by natural selection to observed population structure, we performed additional multivariate analyses using a dataset limited to *F*_ST_ outlier loci. We also performed multivariate analyses using a subset of putatively neutral loci that contributed the most information to DAPC clustering. These loci were identified by performing DAPC using the full dataset, then scaling locus loadings, and calculating locus rank percentiles for discriminant functions one and two. Loci with rank percentiles ≥98.7% for each discriminant function were selected to produce a set of putatively neutral loci that corresponded with a similar number of markers as those identified in *F*_ST_ outlier analyses. Any *F*_ST_ outlier loci occurring in this putatively neutral set of markers were removed. For both datasets, PCoA and *K*‐means clustering followed by DAPC were performed as described above.

#### 2.4.3 Genetic attributes of striped marlin populations

Populations of striped marlin resolved in multivariate and STRUCTURE analyses were characterized by assessing genetic diversity and the presence of SNPs exhibiting fixed differences among populations (i.e., private alleles) using a dataset in which loci not conforming to the expectations of HWE were removed. Observed and expected heterozygosities were calculated in the R packages *poppR* v2.5.0 (Kamvar, Tabima, & Grünwald, 2014) and *dartR*, respectively. We used the R package *PopGenReport* v3.0.0 (Adamack & Gruber, 2014) to calculate rarefaction allelic richness. *dartR* was used to evaluate populations for the presence of private alleles.

Because inferences of demographic relationships may be biased by loci that deviate from a neutral model of evolution (Beaumont & Nichols, 1996; Luikart et al., 2003), SNPs previously identified as *F*_ST_ outliers were removed prior to calculating pairwise levels of genetic differentiation and population‐level inbreeding coefficients. Levels of genetic differentiation among populations were determined by calculating pairwise measures of *F*_ST_ in Arlequin. Statistical significance of *F*_ST_ values was assessed based on 10,000 permutations and a critical value corrected by a modified false discovery rate (Benjamini & Yekutieli, 2001; Narum, 2006). Inbreeding within populations was evaluated by calculating *F*_IS_ in the R package *diveRsity* v1.9 (Keenan, McGinnity, Cross, Crozier, & Prodöhl, 2013). Confidence intervals (95%) for estimates of *F*_IS_ were calculated based on 10,000 bootstrap iterations.

A putatively neutral dataset was also used to infer the degree of genetic connectivity among populations and to identify populations that serve as sources or sinks (Crowder & Norse, 2008; Howe, Davis, & Mosca, 1991), by calculating directional relative migration rates using the *divMigrate* function (Sundqvist, Keenan, Zackrisson, Prodohl, & Kleinhans, 2016) in the R package *diveRsity*. This approach provides relative bidirectional estimates of gene flow based on measures of genetic differentiation between populations, and is considerably less computationally intensive than maximum‐likelihood or Bayesian methods (e.g., Beerli & Palczewski, 2010; Hey, 2010) for estimating migration rates from large genomic datasets. We performed *divMigrate* calculations using all available measures of genetic differentiation so that the consistency of estimates among metrics could be assessed. Confidence intervals (95%) of relative migration estimates were calculated based on 10,000 bootstrap iterations.

#### 2.4.4 Temporal stability of population structure

To evaluate the temporal stability of allele frequencies within geographically distant regions, we used collections with sample sizes ≥15 individuals per sampling period, and for which temporally spaced collections spanned at least one generation of striped marlin (average generation time estimated to be 4.4 years; Collette et al., 2011). These included striped marlin sampled off Ecuador in the years 1992 (ECU 1992, *n* = 15; Table 1) and 2016 (ECU 2016, *n* = 22), spanning approximately five generations of striped marlin. We also evaluated striped marlin sampled off eastern Australia in the year 1994 (EAUS 1994, *n* = 16) and in the years 2010, 2011, 2012, and 2015 (EAUS 2010–2015, *n* = 19), the latter of which were pooled to facilitate the valuable comparison of sample collections spanning 16–21 years (approximately three to five generations of striped marlin). Multigenerational stability of allele frequencies for temporally spaced sample collections was assessed by performing multivariate analyses (PCoA and DAPC) using the full dataset as described above, except only a scenario of *K* = 2 was evaluated with DAPC for each geographic region. This clustering scenario comprised groups of samples corresponding with each sampling period. *F*_ST_ values were also calculated between temporally spaced collections as described above using a dataset in which loci not conforming to the expectations of HWE and selective neutrality were removed.

#### 2.4.5 Population assignment

We assessed the ability of SNPs resolved in this study to accurately assign individuals to populations and to identify a minimum subset of loci for population assignment, using the R package *assigner* (Gosselin, Benestan, & Bernatchez, 2015). Assignment analyses were performed using a dataset in which loci not conforming to the expectations of HWE were removed. The methodological approach described by Anderson (2010) was implemented by randomly selecting 80% and 20% of samples from each population for training and holdout datasets, respectively. Training samples were used to rank loci based on *F*_ST_, and holdout samples were assigned to populations using DAPC as implemented in *adegenet*. Within DAPC assignment analyses, the function *predict.dapc* was used to predict posterior membership probabilities to each population for each individual. We evaluated scenarios where 100–1,000 markers (in increments of 100) were used for population assignment. Analyses were repeated five times for each marker subset, and final results were produced by averaging across iterations.

## 3 RESULTS

### 3.1 SNP quality filtering

The original DArT PL dataset consisted of 61,908 SNP loci (Table 2). A total of 4,206 SNPs remained after quality filtering based on percent missing genotype calls, average reproducibility, monomorphic loci, the presence of more than one SNP per read alignment, and minor allele frequencies <0.05 across all samples. Four samples were missing genotype calls at ≥20% of loci and were excluded from further analyses. Collectively, these filtering steps resulted in a dataset comprising 245 individuals (Table 1) genotyped across 4,206 SNPs (Table 2). This dataset is hereafter referred to as the “full dataset.”

**Table 2:** Number of single nucleotide polymorphism (SNP) loci retained after each filtering step

Filter	No. of loci retained
Loci received from Diversity Arrays Technology Pty. Ltd.	61,908
Quality filter	
Loci missing ≥ 10% genotypes	41,613
Samples missing ≥ 20% genotypes	11,896
Locus average reproducibility < 95%	11,831
Monomorphic loci	11,831
More than one SNP per read alignment	10,220
Locus minor allele frequency < 0.05	4,206
Hardy–Weinberg equilibrium	
*p* < .006 in more than one sample collection	4,165
Outlier identification	
Putatively neutral	4,106
Putatively adaptive	59

### 3.2 Identification of genetically distinct populations

**Figure 2:**
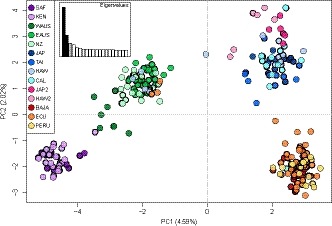
Axes one and two resulting from principal coordinate analysis (PCoA) of the full dataset (*n* = 4,206 SNPs). Percentage of total variation explained by each axis is shown. Sample collections are labeled as in Table 1 and colored according to the legend. Similar colors are used to highlight regional populations. Inset at top left shows eigenvalues associated with the PCoA; black bars correspond with plotted axes

Multivariate analyses and Bayesian simulations were used to delineate populations of striped marlin using the full dataset and a dataset in which loci not conforming to the expectations of HWE were removed, respectively. At least five distinct clusters were resolved on PCoA axes one and two, which collectively explained 6.61% of total genetic variation (Figure 2). These clusters corresponded with striped marlin from the eastern central Pacific Ocean (BAJA, ECU, and PERU), North Pacific Ocean (JAP, TAI, HAW, and CAL), western South Pacific Ocean (EAUS and NZ), eastern Indian Ocean (WAUS), and western Indian Ocean (SAF and KEN). These clusters were also resolved on additional PCoA axes (Figures S1 and S2). Eight striped marlin grouped with collections geographically distant from their sampling location, including four fish sampled off Hawaii and three fish sampled off Ecuador that clustered with striped marlin from the western South Pacific Ocean (EAUS and NZ). Similarly, one fish sampled off California grouped with striped marlin collected from the eastern central Pacific Ocean (BAJA, ECU, and PERU). These eight individuals are presumed to reflect movements between geographically distant regions and are hereafter referred to as putative migrants.

**Figure 3:**
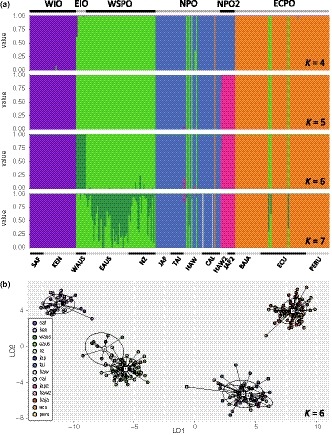
Results from discriminant analysis of principal components (DAPC) using the full dataset (*n* = 4,206 SNPs). (a) Bar plots colored to show posterior probabilities of assignment to a cluster. Scenarios for *K* equal to four through seven are shown. Horizontal bar at bottom delineates sample collections labeled as in Table 1. Horizontal bar at top delineates clusters corresponding with regional populations. (b) Scatter plot of discriminant functions one and two for scenario with *K* equal to six from (a). Samples are colored according to the legend. Inertia ellipses for each group are also shown

Results from DAPC of SNP genotypes revealed hierarchical relationships among multiple genetically distinct groups of striped marlin. BICs generated for *K* values of one through twelve ranged from 1,279.44 to 1,307.55 and were lowest for the scenario with *K* equal to two (BIC = 1,274.45), but were also low for *K* equal to three through six (BIC = 1,275.47–1,283.90). In the scenario with *K* equal to two, one cluster comprised all Indian Ocean collections plus EAUS and NZ, and a second cluster comprised all remaining Pacific Ocean collections. The scenario with *K* equal to three also resolved a cluster comprising EAUS, NZ, and Indian Ocean collections, as well as clusters corresponding with striped marlin from the North Pacific Ocean (JAP, TAI, HAW, and CAL) and from the eastern central Pacific Ocean (BAJA, ECU, and PERU). These clusters were again resolved in the scenario with *K* equal to four (Figure 3a), except striped marlin from Oceania (WAUS, EAUS, and NZ) formed a cluster distinct from western Indian Ocean collections (SAF and KEN). These four clusters were resolved in the scenario with *K* equal to five, except a subset of fish sampled off Japan (*n* = 6) and Hawaii (*n* = 6; hereafter referred to as JAP2 and HAW2, respectively) comprised a fifth cluster also apparent in results from PCoA (Figures 2, S1 and S2). For *K* equal to six, an additional cluster corresponding with the eastern Indian Ocean (WAUS) was resolved, though in some iterations of *K*‐means clustering this cluster was not apparent until *K* increased to seven (Figure S3). Scenarios corresponding with larger *K* values resulted in high degrees of admixture within groups, indicating overfitting of the DAPC statistical model (Figure 3). For each of the scenarios described here, DAPC produced clusters that were clearly resolved (i.e., nonoverlapping) in two‐dimensional plots (Figures 3b and S3b), except for the cluster comprising JAP2 and HAW2, which partially overlapped with the cluster containing remaining collections from the North Pacific Ocean (JAP, TAI, HAW, and CAL). Across *K* scenarios, the single putative migrant sampled off California consistently assigned to the cluster corresponding with striped marlin from the eastern central Pacific Ocean (BAJA, ECU, and PERU). The four putative migrants sampled off Hawaii and three putative migrants sampled off Ecuador consistently assigned to the cluster comprising striped marlin from the western South Pacific Ocean (EAUS and NZ).

The groups of striped marlin resolved in multivariate analyses were also apparent in results from STRUCTURE. Testing of SNPs for conformance to HWE identified 41 loci that violated the expectations of HWE in more than one sample collection; these loci were excluded from STRUCTURE analyses. For STRUCTURE analyses performed with all sample collections, multiple distinct clusters were consistently resolved across scenarios of *K* equal to two through seven (Figure S4). These included a cluster corresponding with striped marlin from the western Indian Ocean (SAF, KEN) characterized by low degrees of shared ancestry with striped marlin from the Pacific Ocean. A second cluster comprising striped marlin from Oceania (WAUS, EAUS, NZ) was also evident. This cluster corresponded with high degrees of shared ancestry with striped marlin from elsewhere in both the Pacific and Indian oceans. Clusters representing striped marlin from the eastern central Pacific Ocean and the North Pacific Ocean were also apparent and were characterized by low degrees of shared ancestry with Indian Ocean fish. The putative migrants sampled off Hawaii and Ecuador displayed admixture proportions consistent with striped marlin from Oceania (Figure S4). Results from Structure Harvester indicated that the most likely *K* for this dataset was five. Examination of Q values estimated in the *K* equal five scenario revealed that in addition to distinct groups corresponding with the western Indian Ocean, Oceania, North Pacific Ocean, and eastern central Pacific Ocean, a fifth group corresponded with a subset of striped marlin from the North Pacific Ocean (JAP2, HAW2).

Similar groups of striped marlin were resolved in STRUCTURE analyses performed on datasets where highly differentiated sample collections were excluded. STRUCTURE analyses limited to striped marlin from the Pacific Ocean and eastern Indian Ocean revealed distinct clusters corresponding with Oceania (WAUS, EAUS, and NZ), the eastern central Pacific Ocean (BAJA, ECU, and PERU), and two regions of the North Pacific Ocean (JAP, TAI, HAW, CAL, JAP2, and HAW2; Figure S5b). Structure Harvester identified the most likely *K* for this dataset as four. STRUCTURE analyses limited to samples collected from the Indian Ocean and western South Pacific Ocean resolved at least two distinct clusters (Figure S5a). Two of these clusters corresponded with the western Indian Ocean (SAF and KEN) and western South Pacific Ocean (EAUS and NZ). Admixture proportions for striped marlin from the eastern Indian Ocean (WAUS) were intermediate to these clusters, and Structure Harvester identified the most likely *K* for this dataset as three. Results from STRUCTURE analyses performed without an admixture model of ancestry are included in the Appendix S1 (Figures S6 and S7).

We compared results from multivariate and STRUCTURE analyses to inform the grouping of sample collections into larger regional assemblages representing genetically distinct populations of striped marlin. Distinct clusters comprising striped marlin from the western Indian Ocean (WIO; sample collections SAF and KEN), eastern Indian Ocean (EIO; sample collection WAUS), western South Pacific Ocean (WSPO; sample collections EAUS and NZ), and eastern central Pacific Ocean (ECPO; sample collections BAJA, ECU, and PERU) were resolved by PCoA, DAPC, and STRUCTURE analyses, and these regions were recognized as distinct populations in subsequent analyses. Striped marlin from the North Pacific Ocean (NPO; sample collections JAP, TAI, HAW, and CAL) were also consistently identified as genetically distinct, as was a second group in the North Pacific Ocean (NPO2) corresponding with JAP2 and HAW2. Population‐level calculations were performed using these six populations of striped marlin. The eight fish identified as putative migrants were retained with their original sample collections so that biologically realistic assemblages of striped marlin could be characterized; however, some calculations were performed a second time with these samples excluded (described below).

### 3.3 SNPs putatively influenced by natural selection

From the 4,165 SNPs remaining after quality filtering and HWE testing, a genome scan performed using BayeScan identified 61 loci (1.46%) as outliers putatively under the influence of natural selection (Figure S8). *F*_ST_ values associated with these SNPs ranged from 0.086 to 0.461, and all loci were candidates for divergent selection (*α* = .49–2.97). Results from outlier detection analyses performed using Arlequin included the identification of 229 SNPs (5.49%) as putatively under selection (Figure S9). Of those loci, 74 were candidates for balancing selection (per locus *F*_ST_ no different from zero) and 155 were candidates for directional selection (per locus *F*_ST_ = 0.089–0.679). Fifty‐nine of the SNPs identified as *F*_ST_ outliers by BayeScan were identified by Arlequin as SNPs likely experiencing directional selection; these SNPs comprised a final list of outlier loci. Six of these loci produced BLASTn hits with expect values <10^–10^, but annotations for specific genes or gene functions were not returned (results not shown).

**Figure 4:**
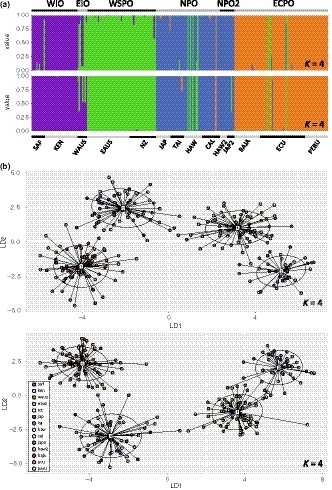
Results from discriminant analysis of principal components (DAPC) using datasets limited to a subset of putatively neutral loci (*n* = 46 SNPs; Panels a and b *top*) or loci putatively influenced by natural selection (*n* = 59 SNPs; Panels a and b *bottom*). Results from *K* equal to four are shown. (a) Bar plots colored to show posterior probabilities of assignment to a cluster. Horizontal bar at bottom delineates sample collections labeled as in Table 1. Horizontal bar at top delineates clusters corresponding with regional populations. (b) Scatter plots of discriminant functions one and two. Samples are colored according to the legend. Inertia ellipses for each group are also shown

Multivariate analyses performed using a dataset limited to SNPs putatively under the influence of natural selection (*n* = 59) resolved populations that displayed comparatively large degrees of genetic differentiation based on the full dataset (WIO, WSPO, NPO, and ECPO), but failed to resolve weakly differentiated populations (EIO and NPO2; Figure 4; results from PCoA not shown). A total of 96 SNPs corresponded with rank percentiles ≥98.7% for DAPC discriminant functions one and two based on results from the full dataset. Fifty of these loci were previously identified as *F*_ST_ outliers and excluded from multivariate analyses performed using remaining putatively neutral markers (*n* = 46). These analyses resolved populations that were separated by comparatively large degrees of genetic differentiation based on the full dataset (WIO, WSPO, NPO, and ECPO; Figure 4).

### 3.4 Genetic attributes of striped marlin populations

To characterize basic genetic attributes of the striped marlin populations resolved in this study, we calculated population‐level diversity and assessed populations for the presence of private alleles. Genetic diversity metrics were calculated using the dataset in which loci not conforming to the expectations of HWE were removed (*n* = 4,165 SNPs). Rarefaction allelic richness and expected heterozygosity were highest for NPO2 (*a*_R_ = 1.410, *H*_E_ = 0.199; Table 3) and lowest for EIO (*a*_R_ = 1.321, *H*_E_ = 0.148); these populations correspond with the smallest sample sizes employed in this study (*n* = 8 and 12 samples, respectively). Measures of genetic diversity for EIO were comparable to those for WIO (*a*_R_ = 1.332, *H*_E_ = 0.149), which comprised a larger sample size (*n* = 38). This result indicates that the low levels of genetic diversity observed for EIO may reflect low genetic diversity for striped marlin across the Indian Ocean, rather than an artifact of the small sample size for this population. High levels of genetic diversity estimated for NPO2 may accurately reflect elevated genetic heterogeneity for this population, including relative to NPO (*a*_R_ = 1.355, *H*_E_ = 0.158); however, a larger number of samples for NPO2 are necessary to evaluate this hypothesis. Additional samples for this population are also necessary to assess mechanisms underlying the large difference between observed and expected heterozygosities (*H*_O_ = 0.293, *H*_E_ = 0.199). We did not observe any private alleles indicating fixed differences between populations, regardless of whether putative migrants were excluded from calculations. Genetic diversity metrics were also calculated for each sampling location (Table S1).

**Table 3:** Genetic metrics calculated for striped marlin (*Kajikia audax*) populations resolved in this study

Population	Sample collections	*N*	*a*_R_	*H*_E_	*H*_O_	*F*_IS_	*F*_IS_ CI
WIO	SAF, KEN	38	1.332	0.149	0.143	0.060	0.040–0.064
EIO	WAUS	8	1.321	0.148	0.137	0.060	−0.078–0.052
WSPO	EAUS, NZ	57	1.356	0.16	0.164	0.013	−0.015–0.025
NPO	JAP, TAI, HAW, CAL	53	1.355	0.158	0.157	0.068	0.042–0.080
NPO2	JAP2, HAW2	12	1.410	0.199	0.293	−0.404	−0.519– −0.374
ECPO	BAJA, ECU, PERU	77	1.349	0.157	0.160	0.041	0.017–0.054

Abbreviations: *a*_R_, rarefaction allelic richness; *F*_IS_, inbreeding coefficient; *F*_IS_ CI, inbreeding coefficient 95% confidence intervals; *H*_E_, expected heterozygosity; *H*_O_, observed heterozygosity; *N*, population sample size.

Note: Values for diversity metrics are colored as a heat map where darker colors correspond with higher values.

**Figure 5:**
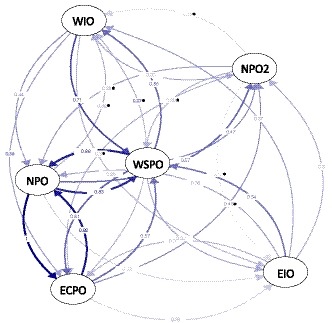
Bidirectional relative migration rates among striped marlin (*Kajikia audax*) populations calculated using a dataset where loci not conforming to Hardy–Weinberg equilibrium and selective neutrality were removed (*n* = 4,106 SNPs). Open circles represent populations and lines connecting circles are weighted according to relative migration rate. Relative migration rates with 95% confidence intervals larger than 0.00 are denoted with an asterisk. Values shown here were calculated with putative migrants included

Loci previously found to deviate from a neutral model of evolution (*n* = 59; described above) were excluded to produce a putatively neutral dataset prior to calculating pairwise measures of genetic differentiation, inbreeding coefficients, and relative migration rates. Pairwise *F*_ST_ values ranged from 0.0137 between EIO and WSPO, to 0.0819 between WIO and NPO2 (Table 4). All *F*_ST_ values were statistically significant at *p* = .000. Pairwise *F*_ST_ values were also calculated for sampling locations (Table S2). Inbreeding coefficients ranged from −0.404 (95% CI = −0.519 to −0.374) in NPO2 to 0.068 (95% CI = 0.042–0.080) in NPO, indicating negligible levels of inbreeding within populations of striped marlin (Table 3). Relationships of genetic connectivity among populations inferred by calculating bidirectional relative migration rates (*m*_R_) were similar across all three metrics of genetic differentiation (Jost's D, G_ST_, and N_M_; Alcala, Goudet, & Vuilleumier, 2014; Jost, 2008; Nei, 1973), except the magnitude of these relationships was lower for calculations based on Jost's D. We therefore describe results for only one of these metrics (N_M_; Figure 5). The largest relative migration rates (*m*_R_ > 0.90) corresponded with gene flow in both directions between ECPO and NPO. High levels of genetic connectivity were also observed in both directions between NPO and WSPO (*m*_R_ > 0.80), and from WIO to WSPO (*m*_R_ = 0.71). Relative migration rates between WIO and EIO (*m*_R_ = 0.28 for WIO to EIO; *m*_R_ = 0.37 for EIO to WIO) were lower than migration rates between most Pacific Ocean populations. Additionally, relative migration rates between WIO and WSPO (*m*_R_ ≥ 0.58) were higher than those between EIO and WSPO (*m*_R_ ≤ 0.54), despite the closer geographic proximity of EIO to WSPO. Accurate inference of relative migration rates is difficult under scenarios of high gene flow (Sundqvist et al., 2016); this may be particularly so for EIO and WSPO, especially given the small sample size for EIO (*n* = 8). Migration rates calculated between Indian Ocean and Pacific Ocean populations of striped marlin indicate that genetic connectivity between ocean basins is primarily facilitated by WSPO. Relative migration rates calculated with putative migrants excluded from analyses produced relationships similar to those described here (Figure S10).

**Table 4:** Pairwise *F*_ST_ values (below diagonal) and corresponding *p*‐values (above diagonal) calculated between striped marlin (*Kajikia audax*) populations resolved in this study

	WIO	EIO	WSPO	NPO	NPO2	ECPO
WIO	–	0.000	0.000	0.000	0.000	0.000
EIO	0.0241	–	0.000	0.000	0.000	0.000
WSPO	0.0284	0.0137	–	0.000	0.000	0.000
NPO	0.0516	0.0329	0.0214	–	0.000	0.000
NPO2	0.0819	0.0707	0.0523	0.0361	–	0.000
ECPO	0.0614	0.0480	0.0359	0.0191	0.0524	–

Note

*F*_ST_ values are colored as a heat map where darker colors correspond with higher values. *p*‐values associated with each pairwise comparison are shown above diagonal.

### 3.5 Temporal stability of population structure

**Figure 6:**
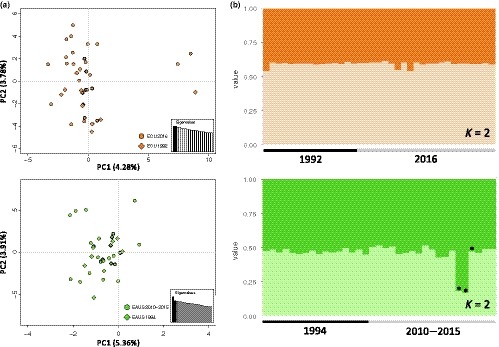
Results from principal coordinate analysis (PCoA; a) and discriminant analysis of principal components (DAPC; b) for temporal collections comprising striped marlin (*Kajikia audax*) sampled off Ecuador (top row; years 1992 and 2016) and eastern Australia (bottom row; years 1994 and 2010–2015). Analyses were performed using the full dataset (*n* = 4,206 SNPs). (a) Plot of PCoA axes one and two. Percentage of total variation explained by each axis is shown. Temporal collections are distinguished by plotting symbols (circles, diamonds). Insets show eigenvalues associated with the PCoA; black bars correspond with plotted axes. (b) Bar plots colored to show posterior probabilities of assignment to one of two clusters defined according to sampling period. Asterisks distinguish striped marlin sampled off eastern Australia in 2016 (*n* = 3)

We assessed the multigenerational stability of allele frequencies within two geographically distant regions by comparing striped marlin sampled from the eastern central Pacific Ocean in 1992 and 2016, and from the western South Pacific Ocean in 1994 and 2010–2015, using the full dataset. PCoA of collections from the eastern central Pacific Ocean resolved a large cluster of fish comprising both sampling periods, except for three individuals positioned adjacent to this cluster (Figure 6a). These three samples comprised fish from both the 1992 and 2016 collections. Posterior probabilities of assignment resulting from DAPC for the two groups of striped marlin corresponding with each sampling period were similar across all individuals (Figure 6b). For temporally spaced collections from the western South Pacific Ocean, PCoA resolved a single cluster of striped marlin; however, DAPC performed for groups corresponding with each sampling period revealed two striped marlin with assignment probabilities distinct from other fish. These two individuals were sampled in 2012, but a third fish sampled in the same year displayed assignment probabilities consistent with striped marlin from all other years.

Genetic differentiation calculated between temporally spaced collections using a dataset in which loci not conforming to the expectations of HWE and selective neutrality were removed resulted in *F*_ST_ values that were low but statistically significant for both geographic regions (eastern central Pacific: *F*_ST_ = 0.0029, *p* = .002; western South Pacific: *F*_ST_ = 0.0042, *p* = .000). These levels of genetic differentiation are considerably lower than those observed for population‐level comparisons (*F*_ST_ = 0.0137–0.0819), in several instances by a full order of magnitude. Additionally, multivariate analyses performed with all samples resolved temporally spaced collections as comprising single groups within the eastern central Pacific and western South Pacific oceans. Collectively, results from comparisons of temporal collections are consistent with the stability of allele frequencies for a minimum of three to five generations of striped marlin in the eastern central Pacific and western South Pacific oceans.

### 3.6 Population assignment

**Figure 7:**
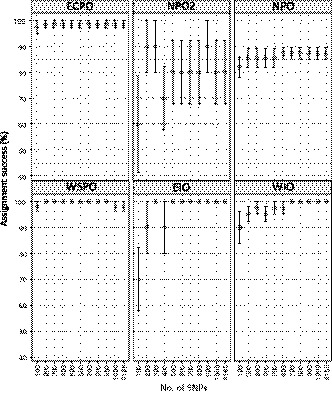
Average assignment accuracy of striped marlin (*Kajikia audax*) individuals to populations based on locus subsets comprising 100–1,000 SNPs. Results from tests performed using the full dataset (*n* = 4,165 SNPs) are also shown

Overall assignment success was ≥90% (*SE* ± 2.29–4.55) when subsets of ≥200 SNPs were used to assign individuals to populations. Within populations, ≥90% assignment success was possible for ECPO (*SE* ± 1.40–2.60), WSPO (*SE* ± 0.00–1.80), and WIO (*SE* ± 0.00–6.12) across all SNP subsets (Figure 7). Population assignment success for EIO was consistently ≥90% only when larger subsets of loci were used (≥ 500 SNPs). In the North Pacific Ocean, assignment success was comparatively low, ranging from 82% to 87% (*SE* ± 2.20–4.02) for NPO and 60 to 80% (*SE* ± 10.00–18.71) for NPO2. Low assignment success and high standard errors for NPO2 may result from a comparatively high degree of genetic heterogeneity for this population (see results above). A larger sample size for NPO2 is necessary to improve assignment accuracy for this population.

## 4 DISCUSSION

The primary goal of this study was to resolve spatiotemporal patterns of genomic variation across the full range of a broadly distributed and highly migratory large pelagic species, providing practical information for species conservation and fisheries management, and advancing our understanding of genetic connectivity in pelagic environments. To accomplish this goal, we surveyed over 4,000 SNPs in striped marlin sampled across the Pacific and Indian oceans and analyzed resulting data to assess spatial population structure, the presence of genetic variation potentially influenced by natural selection, and the temporal stability of allele frequencies. Individual‐based cluster analyses resolved six genetically distinct populations of striped marlin corresponding with the western Indian, eastern Indian, western South Pacific, and eastern central Pacific oceans, as well as two populations in the North Pacific Ocean. Populations separated by comparatively large degrees of genetic differentiation were resolved using loci putatively under the influence of natural selection or using a subset of putatively neutral loci. Allele frequencies for temporal replicates from the western South Pacific and eastern central Pacific oceans were stable for periods of three to five generations of striped marlin. Collectively, these results demonstrate that species capable of long‐distance dispersal in environments lacking obvious physical barriers to movement can display substantial population subdivision that persists over multiple generations and is putatively facilitated by both neutral and adaptive processes. Our results also highlight substantial inconsistencies between genetically distinct populations of striped marlin and stock boundaries currently recognized by RFMOs.

### 4.1 Biological context of genetically distinct populations

This study represents the first assessment of spatial genetic variation for striped marlin in the Indian Ocean and provides evidence for genetically distinct populations in the western Indian and eastern Indian oceans. Seasonal movements and spawning behavior are poorly understood for striped marlin in the Indian Ocean, but tagging efforts in the western Indian Ocean reflect movements restricted to this region, with the exception of a fish recaptured off Western Australia (Roy Bealey, African Billfish Foundation, personal communication). Striped marlin larvae have been collected from both western and eastern regions of the Indian Ocean during seasons that at least partially overlap between regions (Bromhead, Pepperell, Wise, & Findlay, 2003; Jones & Kumaran, 1964; Nakamura, 1983; Nishikawa, Kikawa, Honma, & Ueyanagi, 1978; Pillai & Ueyanagi, 1978; Ueyanagi, 1974). Our results demonstrate low degrees of shared ancestry and high levels of genetic differentiation between the population of striped marlin in the western Indian Ocean and populations in the Pacific Ocean. In comparison, eastern Indian Ocean striped marlin exhibited a close genetic relationship with striped marlin in the western South Pacific Ocean; genetic differentiation between these regions was also lower than that between Indian Ocean populations. Relative migration rates reflect levels of gene flow from the Indian Ocean to the western South Pacific Ocean that are greater than those in the opposite direction, highlighting the role of the western South Pacific in facilitating genetic connectivity from Indian Ocean to Pacific Ocean populations of striped marlin. Possible mechanisms facilitating interoceanic gene flow include passive drift of eggs and larvae, and dispersal of adult fish. Pelagic larval duration is not known for striped marlin or other closely related species, but can strongly influence population‐level patterns of genetic connectivity in marine systems (Selkoe & Toonen, 2011). Movements of adult striped marlin between the Pacific and Indian oceans have not been reported, although tagging and reporting efforts have been limited in regions across the Indo‐Pacific. The temperate waters typically inhabited by striped marlin (20–25°C sea surface temperature; Howard & Ueyanagi, 1965; Sippel et al., 2007) and occurrence of seasonal assemblages in waters as far south as Tasmania (Bromhead et al., 2003) suggest interoceanic movements of striped marlin around the Australian continent may be possible in at least some years.

Our results clarify previously ambiguous population‐level relationships for striped marlin in the Pacific Ocean and are generally consistent with biological information available for this region. We confirm the presence of two genetically distinct populations of striped marlin in the North Pacific Ocean, one of which corresponds with a subset of fish sampled off Japan and Hawaii. The possibility of two biologically distinct assemblages of striped marlin in waters off Hawaii was initially proposed by Bromhead et al. (2003) based on a bimodal size distribution in catches reported from this region and high seasonal abundances of juvenile fish. A subsequent genetic study reported statistically significant genetic differentiation between reproductively immature and mature striped marlin off Hawaii (Purcell & Edmands, 2011); however, that result was based on microsatellite data corrected for null alleles, and comparisons with uncorrected data were not significant. Incomplete biological information for samples comprising NPO2 prohibits comparisons of demographic characteristics (e.g., size class) between the North Pacific Ocean populations resolved in this study. Spawning activity has been observed for two geographically distant regions of the North Pacific Ocean (Hyde, Humphreys, Musyl, Lynn, & Vetter, 2006; Sun et al., 2011) and for areas corresponding with additional populations of striped marlin in the Pacific Ocean (Eldridge & Wares, 1974; González‐Armas, Klett‐Traulsen, & Hernández‐Herrera, 2006; Kopf, Davie, Bromhead, & Young, 2012; Kume & Joseph, 1969). Regional movements of striped marlin generally correspond with the genetically distinct populations described here (Domeier, 2006; Holdsworth et al., 2009; Ortiz et al., 2003; Sippel et al., 2007). The putative migrants identified in genomic analyses represent movements between the western South Pacific Ocean and the North Pacific and eastern central Pacific oceans that have not been reported from tagging efforts, demonstrating the utility of genetic information in clarifying ocean‐wide migration patterns. Whether movements of these putative migrants are accompanied by gene flow is not known, but the presence of genetically distinct populations in these regions suggests that genetic connectivity between regions is low, and these fish likely represent vagrants exploiting geographically distant foraging grounds.

Surveys of genome‐wide variation in highly migratory large pelagic fishes with spatial distributions spanning the Pacific or Indian oceans are presently limited, but available studies report patterns of population subdivision similar to those observed here. Analyses of neutral and putatively adaptive markers identified from over 7,000 SNPs in the near‐threatened Galapagos shark (*Carcharhinus galapagensis*) revealed the presence of genetically distinct populations in the western Indian, western South Pacific, central North Pacific, eastern North Pacific, and eastern central Pacific oceans (Pazmiño et al., 2018). For sampling locations represented in both that and the present study, regions corresponding with genetically distinct populations were identical, except we resolved striped marlin sampled from waters off Central America and South America as belonging to the same population. Assessment of over 6,000 SNPs for yellowfin tuna in the Pacific Ocean resolved genetically distinct populations in the western South Pacific, central Pacific, and eastern North Pacific oceans (Grewe et al., 2015), demonstrating regional population structure similar to that observed in the present study. In contrast, analyses of over 1,500 SNPs in black marlin (*Istiompax indica*) identified a single population spanning eastern and western regions of the Indian Ocean; a second population spanning the western South Pacific, central North Pacific, and eastern central Pacific oceans; and a third population in the South China Sea (Williams, 2018). Identifying mechanisms underlying differences in ocean‐wide patterns of genetic connectivity among large pelagic fishes requires improved knowledge of species' biological characteristics (e.g., thermal preferences, dispersal capabilities, degree of fidelity to natal spawning grounds) and sensitivity to obvious or cryptic barriers to movement.

### 4.2 Biological significance of statistically significant comparisons

The populations of striped marlin resolved in this study were separated by levels of genetic differentiation that were also highly statistically significant. However, in some instances, comparatively low levels of genetic differentiation were also statistically significant. Low but statistically significant *F*_ST_ values (*F*_ST_ = 0.0018–0.0065, *p* = .001–.009) were observed for five comparisons between sample collections within a population. An additional statistically significant comparison was observed within NPO2 (*F*_ST_ = 0.0182, *p* = .000), but in this case, the level of genetic differentiation was nearly three times higher than other statistically significant comparisons within populations. Low but statistically significant *F*_ST_ values were also observed within temporal collections (eastern central Pacific: *F*_ST_ = 0.0029, *p* = .002; western South Pacific: *F*_ST_ = 0.0042, *p* = .000). Aside from the comparison within NPO2, *F*_ST_ values associated with statistically significant comparisons within populations or temporal collections were less than half of those calculated between populations of striped marlin. This observation suggests that such low levels of genetic differentiation may not be biologically meaningful and may instead represent random sampling error due to the high level of statistical power associated with surveying a large number of genome‐wide molecular markers, but comparatively low power corresponding with relatively small sample sizes per sample collection.

Genetic differentiation between populations of marine fishes is expected to be lower than in freshwater and anadromous fishes (Ward, Woodwark, & Skibinski, 1994), presumably due to greater opportunities for dispersal in marine environments. Distinguishing biologically meaningful levels of genetic differentiation from stochastic noise is therefore challenging in marine fishes (Waples, 1998) and may be particularly so for species with enhanced dispersal capabilities, such as large pelagic fishes. Under these circumstances, determining whether a statistically significant test result is also of biological significance may be context‐dependent (Waples, 1998). Further exploration of the levels of genetic differentiation expected for varying experimental designs, species life histories, and genome‐wide markers (e.g., Alcala & Rosenberg, 2017; Jost et al., 2018) is necessary for assisting the interpretation of results from genomic studies of broadly distributed and highly migratory large pelagic fishes.

### 4.3 Relative contributions of neutral and adaptive processes to population structure

Population census sizes (N_c_) for many marine fishes are very large relative to terrestrial or freshwater species and may also correspond with comparatively large effective population sizes (N_e_; Laconcha et al., 2015; Waples, Grewe, Bravington, Hillary, & Feutry, 2018; but see Hauser & Carvalho, 2008; Palstra & Ruzzante, 2008). For marine populations with large Ne, accumulation of allele frequency differences between populations due to neutral processes may be slow and counteracted by individuals that stray and reproduce in non‐natal populations. This may be a greater possibility in marine fishes that are broadly distributed in pelagic environments and have high dispersal capabilities. It is therefore likely that additional mechanisms, such as those corresponding with regional selective pressures, contribute to the accumulation of genetic differences among populations of pelagic marine fishes (Hauser & Carvalho, 2008). In this study, we resolved striped marlin populations separated by comparatively high degrees of genetic differentiation using datasets limited to either SNPs putatively under the influence of natural selection or a subset of putatively neutral SNPs. These results indicate that both neutral and adaptive processes may be important in facilitating population subdivision in striped marlin. In comparison, Grewe et al. (2015) resolved Pacific Ocean populations of yellowfin tuna using SNPs putatively under the influence of natural selection (*n* = 215), but those populations were not apparent in analyses based on putatively neutral loci (*n* = 5,054). Further investigation is necessary to characterize mechanisms (e.g., N_e_:N_c_, demographic history, selection strength) underlying the relative capabilities of neutral genomic variation and variation influenced by natural selection to resolve populations of highly migratory large pelagic fishes. Such studies will ultimately inform our presently limited understanding of ecological and environmental variables, phenotypes, and loci involved in regional adaptive processes for large pelagic species.

### 4.4 Sampling designs for studies of large pelagic fishes

For many highly migratory large pelagic fishes of conservation concern, implementing biologically informed sampling designs that target seasonal aggregations across the species range within the same year is complicated by incomplete information on species life histories and limited opportunities to sample pelagic environments. Sampling efforts are further challenged by species that occur at relatively low frequencies. These difficulties typically result in experimental designs where species are opportunistically sampled across several years, producing sample collections that may be small, represent only a portion of the species range, or lack sizeable temporal replicates. The opportunistic sampling design implemented in this study facilitated genomic evaluation of striped marlin across the full species range; however, sample sizes for some populations (EIO and NPO2) are small and may contribute uncertainty to estimates of population‐level relationships (e.g., genetic diversity, migration rates, population assignment). Similarly, temporal replicates for striped marlin from some locations (EAUS and ECU) facilitated evaluation of the multigenerational stability of allele frequencies for populations in these regions (WSPO and ECPO). However, a lack of replicate collections for other regions prohibited exploration of temporal patterns for additional populations. Though these limitations reflect persistent challenges to implementing biologically representative sampling designs for large pelagic fishes, surveys of genome‐wide variation enable robust assessments of population‐level relationships using sample sizes that are more tractable than those required of traditional markers. Additionally, results from genomic surveys such as that implemented in this study can inform targeted spatiotemporal sampling efforts for future studies of large pelagic fishes.

### 4.5 Implications for species conservation and fisheries management

A prerequisite for sustainably managing wild populations is delineating management units that correspond with biologically distinct assemblages (Reiss, Hoarau, Dickey‐Collas, & Wolff, 2009) and should therefore represent a primary goal of fisheries management. Failure to recognize biologically distinct populations in management plans can result in decreased N_e_ and corresponding losses of genetic diversity (Allendorf, Berry, & Ryman, 2014; Allendorf, England, Luikart, Ritchie, & Ryman, 2008; Gaggiotti & Vetter, 1999; Pinsky & Palumbi, 2014), leading to reduced overall productivity of fish stocks and the depletion or loss of more vulnerable populations (e.g., those characterized by lower levels of recruitment). However, current management units for several actively managed marine fishes are primarily based on socioeconomic and political factors rather than species biology (Campana, 2016; Pons, Melnychuk, & Hilborn, 2018). Identifying strategies to balance pragmatic constraints on fisheries management with information derived from genetic and other scientific studies is increasingly important for promoting the long‐term sustainability of wild populations (Garner et al., 2016; Ovenden, Berry, Welch, Buckworth, & Dichmont, 2015; Waples, Punt, & Cope, 2008).

**Figure 8:**
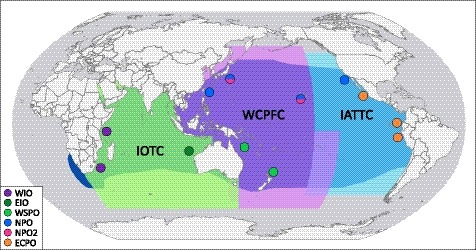
Genetically distinct populations of striped marlin (*Kajikia audax*) overlaid with spatial jurisdictions of regional fisheries management organizations for highly migratory large pelagic fishes (Indian Ocean Tuna Commission [IOTC], Western and Central Pacific Fisheries Commission [WCPFC], and Inter‐American Tropical Tuna Commission [IATTC]). Circles correspond with striped marlin sampling locations and are colored by population; circles composed of two colors represent regions utilized by two populations

In the case of striped marlin, considerable mismatch exists between stocks recognized for fisheries management and what is known regarding the life history and spatial genetic structure of this species (Figure 8). In the Pacific Ocean, three management units corresponding with the western South Pacific, western and central North Pacific, and eastern Pacific oceans are currently recognized. Results from this study indicate that changes to the number and spatial extent of management units in the North Pacific Ocean are necessary, and are especially important given that striped marlin are considered overfished and experiencing unsustainable levels of fishing effort in this region (WCPFC, 2018). Additionally, fisheries in the central North Pacific and western North Pacific (at least in waters off Hawaii and Japan) may seasonally interact with more than one stock, and approaches that account for mixed‐stock fisheries (Crozier et al., 2004) are necessary in these regions. In the Indian Ocean, management units corresponding with the eastern Indian and western Indian oceans should be implemented and are critical given the excessive levels of fishing effort and heavily overfished status estimated for striped marlin in this region (IOTC, 2017). These efforts are also important given the comparatively low levels of genetic diversity observed for Indian Ocean populations in this study and the relative genetic isolation of the western Indian Ocean compared with other populations. Collectively, these changes will reduce mismatches between biologically distinct populations of striped marlin and stocks recognized for fisheries management, improving the effectiveness of management efforts for this commercially and recreationally important species. Implementation of such efforts may be assisted by genetic stock identification (e.g., Larson, Seeb, Pascal, Templin, & Seeb, 2014; McKinney, Seeb, & Seeb, 2017) using the informative subsets of SNPs identified through assignment analyses in this study.

Results from this and other similar studies equip resource managers to incorporate information on genomic variation into evolutionary‐based management plans for highly migratory large pelagic fishes. Preservation of both neutral and adaptive genomic variation is essential to maintaining the adaptive potential of populations and their ability to withstand fluctuations in ecological and environmental conditions (Funk, Forester, Converse, Darst, & Morey, 2018; Funk et al., 2012). Characterizing spatiotemporal patterns of genetic connectivity in pelagic species is also a necessary first step toward enabling spatially explicit investigations to identify drivers of genomic variation in pelagic systems. Such seascape genomic studies (Grummer et al., 2019; Selkoe et al., 2016) have lagged behind that in terrestrial systems, limiting our understanding of demogenetic and evolutionary processes dominating marine environments. Combined with continually improving geospatial resources, newly developed genomic resources will facilitate predictions of the impacts of future ecological and environmental conditions on population health and adaptive potential (Grummer et al., 2019). Information resulting from such studies will further enable evolutionary‐based management efforts that promote resilient populations of highly migratory large pelagic fishes.

### 4.6 Future directions and concluding remarks

This and other recent surveys of genome‐wide variation in highly migratory large pelagic fishes present some of the first genomic resources for these species, particularly for those found at comparatively lower frequencies or of lesser commercial value. Results from this study provide evidence for six genetically distinct populations of striped marlin in the Indo‐Pacific and are valuable for improving rangewide conservation and management efforts for this species. Additional work that employs fine‐scale spatiotemporal sampling is necessary for identifying regional stock boundaries and whether these boundaries are seasonally dynamic. Such efforts will also help determine the contributions of distinct stocks to mixed‐stock fisheries. Studies that implement tagging technology capable of monitoring detailed movements over periods longer than 1 year are presently lacking, but necessary for elucidating seasonal movement patterns within and between ocean basins, and for determining the degree to which populations of striped marlin display spawning site fidelity. Equivalent genomic and tagging efforts across large pelagic fishes will provide valuable insights into broad‐scale patterns of connectivity within and between ocean basins, and ecological and evolutionary factors influencing genetic connectivity in pelagic communities (Hand, Lowe, Kovach, Muhlfeld, & Luikart, 2015; Raeymaekers et al., 2017). The genomics era represents an important opportunity to provide novel information that improves management and conservation initiatives for wild populations (Meek & Larson, 2019), including those of large pelagic fishes, promoting the sustainable use of these ecologically and economically valuable resources.

## CONFLICT OF INTEREST

None declared.

## Data Availability

Data available from the Dryad Digital Repository: https://doi.org/10.5061/dryad.3j9kd51cp (Mamoozadeh, Graves, & McDowell, 2019).
