# Supplementary material for: Genome‐wide SNPs resolve spatiotemporal patterns of connectivity within striped marlin (Kajikia audax), a broadly distributed and highly migratory pelagic species
Source: Evol Appl. 2019 Nov 22;13(4):677–98. doi: 10.1111/eva.12892 (PMC7086058; doi:10.1111/eva.12892)
Supplement: Supplementary file 1 [file EVA-13-677-s001.docx]

**APPENDIX S1**

**Supplemental Information**

**1. DArTseq^TM^ 1.0 genotyping**

DArTseq^TM^ genotyping (Sansaloni et al., 2011) involves genomic complexity reduction followed by NGS, and is similar to other commonly utilized approaches for NGS of reduced genomic representations (e.g. double digest restriction associated DNA sequencing; Peterson, Weber, Kay, Fisher, & Hoekstra, 2012). Genomic complexity reduction was principally performed as described in Kilian et al. (2012), but with a double restriction enzyme (RE) digestion and ligation with RE-specific adapters. Four RE combinations were tested at the Diversity Arrays Technology Pty. Ltd. (DArT PL; Canberra, Australia) facility and digestion with *Pst*I and *Sph*I was selected based on the size of the representation and the fraction of the genome selected. Custom proprietary adapters used in ligation reactions were similar to those described by Elshire et al. (2011) and (Kilian et al., 2012). A *Pst*I-compatible forward adapter included an Illumina flowcell attachment sequence, a sequencing primer sequence, and a variable length barcode. A *Sph*I-compatible reverse adapter included an Illumina flowcell attachment region. Following double RE digestion and adapter ligation, fragments with *Pst*I-*Sph*I overhangs were preferentially amplified in PCR reactions using the following conditions: initial denaturation at 94 ˚C for 1 min, 30 cycles of 94 ˚C for 20 sec, 58 ˚C for 30 sec, and 72 ˚C for 45 sec, and a final extension at 72 ˚C for 7 min. PCR amplification products were subsequently cleaned using a GenElute PCR Clean-Up Kit (Sigma-Aldrich) and visualized on 0.8% agarose gels. Samples for which RE digestion appeared to be incomplete or PCR amplification was unsuccessful were excluded from further library preparation. Samples were normalized and pooled at equimolar ratios into multiplex libraries each comprising 94 samples and two controls, and sequenced for 77 cycles of single-end sequencing on single lanes of an Illumina HiSeq 2500 platform (Illumina, Inc.) at the DArT PL facility.

Raw Illumina reads were processed in CASAVA v1.8.2 (Illumina, Inc.) for initial assessment of read quality and sequence representation, and to produce FASTQ output files. Resulting FASTQ files were analyzed in the proprietary DArTseq^TM^ analytical software pipeline DArTtoolbox, wherein quality filtering, variant calling, and generation of final genotypes were performed in sequential primary and secondary workflows. In the primary workflow, reads with Q < 25 for at least 50% of bases were removed, followed by the removal of reads with Q < 30 in the barcode region. Reads were de-multiplexed according to sample-specific barcodes, then queried against catalogued sequences in the NCBI GenBank and proprietary DArTdb databases to identify and remove reads associated with viral or bacterial contamination. In the secondary workflow, a catalog of reduced representation loci (RRL) was created *de novo* by first aligning identical reads within and among sequenced individuals to form read clusters. Read clusters were catalogued in DArTdb then matched against each other based on degree of similarity and size to form RRL. Polymorphic positions within RRL were distinguished as SNP variants, and major and alternate alleles for each variant were identified. Robust variant calling was ensured by removing SNP loci that met any of the following conditions: monomorphic clusters, clusters containing tri-allelic or aberrant SNPs, clusters with overrepresented sequences, and/or loci lacking both homozygote and heterozygote allelic states. A proportion of loci were produced a second time to assess technical replication error. Each remaining SNP locus was then characterized by calculating major and alternate allele frequency, heterozygote and homozygote frequency, polymorphism information content, call rate, and average reproducibility. DArT PL supplied a final genotype matrix of SNP loci and metadata associated with each locus.

**2. Literature Cited**

Elshire, R., Glaubitz, J., Sun, Q., Poland, J., Kawamoto, K., Buckler, E., & Mitchell, S. (2011). A robust, simple genotyping-by-sequencing (GBS) approach for high diversity species. *PloS ONE*, *6*, e19379. https://doi.org/10.1371/journal.pone.0019379

Kilian, A., Wenzl, P., Huttner, E., Carling, J., Xia, L., Caig, V., … Uszynski, G. (2012). Diversity arrays technology: a generic genome profiling technology on open platforms. In F. Pompanon & A. Bonin (Eds.), *Data production and analysis in population genomics: methods and protocols* (Vol. 888, pp. 67–89). New York, New York, United States of America: Humana Press. https://doi.org/10.1007/978-1-61779-870-2

Peterson, B., Weber, J., Kay, E., Fisher, H., & Hoekstra, H. (2012). Double digest RADseq: an inexpensive method for de novo SNP discovery and genotyping in model and non-model species. *PloS ONE*, *7*, e37135. https://doi.org/10.1371/journal.pone.0037135

Sansaloni, C., Petroli, C., Jaccoud, D., Carling, J., Detering, F., Grattapaglia, D., & Kilian, A. (2011). Diversity Arrays Technology (DArT) and next- generation sequencing combined: genome-wide, high throughput, highly informative genotyping for molecular breeding of Eucalyptus. *BMC Proceedings*, *5 Suppl 7,* P54. https://doi.org/10.1186/1753-6561-5-S7-P54

**Table S1.** Diversity metrics calculated for collections of striped marlin (*Kajikia audax*) sampled from geographically distant regions. Values for diversity metrics are colored as a heat map where darker colors correspond with higher values. Sample collections are labeled as in Table 1.

| Sample Collection | N | a_R_ | H_E_ | H_O_ |
| --- | --- | --- | --- | --- |
| SAF | 11 | 1.261 | 0.146 | 0.138 |
| KEN | 27 | 1.270 | 0.149 | 0.142 |
| WAUS | 8 | 1.263 | 0.148 | 0.137 |
| EAUS | 35 | 1.294 | 0.163 | 0.173 |
| NZ | 22 | 1.279 | 0.155 | 0.147 |
| JAP | 12 | 1.273 | 0.152 | 0.140 |
| JAP2 | 6 | 1.313 | 0.183 | 0.252 |
| TAI | 11 | 1.278 | 0.156 | 0.146 |
| HAW | 15 | 1.290 | 0.161 | 0.164 |
| HAW2 | 6 | 1.376 | 0.221 | 0.330 |
| CAL | 15 | 1.285 | 0.157 | 0.149 |
| BAJA | 21 | 1.275 | 0.153 | 0.147 |
| ECU | 37 | 1.291 | 0.161 | 0.165 |
| PERU | 19 | 1.275 | 0.153 | 0.144 |

N = location sample size

a_R_ = rarefaction allelic richness

H_E_ = expected heterozygosity

H_O_ = observed heterozygosity

**Table S2.** Pairwise F_ST_ values (below diagonal) calculated between collections of striped marlin (*Kajikia audax*) sampled from geographically distant regions. F_ST_ values are colored as a heat map where darker colors correspond with higher values. P-values associated with each pairwise comparison are shown above diagonal. Comparisons with p-values greater than a corrected critical value of 0.010 are marked with an asterisk. Sample collections are labeled as in Table 1.

**Figure S1.** Axes one and three resulting from principal coordinate analysis (PCoA) of the full dataset (n = 4,206 SNPs). Percentage of total variation explained by each axis is shown. Sample collections are labeled as in Table 1 and colored according to the legend. Similar colors are used to highlight regional populations. Inset at top left shows eigenvalues associated with the PCoA, black bars correspond with plotted axes.

**Figure S2.** Axes two and three resulting from principal coordinate analysis (PCoA) of the full dataset (n = 4,206 SNPs). Percentage of total variation explained by each axis is shown. Sample collections are labeled as in Table 1 and colored according to the legend. Similar colors are used to highlight regional populations. Inset at top left shows eigenvalues associated with the PCoA, black bars correspond with plotted axes.

**Figure S3.** Results from discriminant analysis of principal components (DAPC) using the full dataset (n = 4,206 SNPs). A genetically distinct group corresponding with the eastern Indian Ocean (EIO) is not apparent until K equal seven. **Panel** **A:** Bar plots colored to show posterior probabilities of assignment to a cluster. Vertical bars correspond with samples. Scenarios for K equal to four through seven are shown. Horizontal bar at bottom delineates sample collections labeled as in Table 1. Horizontal bar at top delineates clusters corresponding with populations. **Panel** **B:** Scatter plot of discriminant functions one and two for scenarios with K equal to six and seven from Panel A. Samples are colored according to the legend. Inertia ellipses for each group are also shown.

**Figure S4.** Barplots displaying admixture proportions inferred from STRUCTURE analyses performed using a dataset including all sample collections and 4,165 SNPs. Results from scenarios with K equal to two through seven are shown. Individuals are ordered identically across panels. Sample collections are shown at bottom of figure and are labeled as in Table 1. Horizontal bar at top corresponds with populations resolved in this study.

**Figure S5.** Barplots displaying admixture proportions inferred from STRUCTURE analyses performed using datasets (n = 4,165 SNPs) limited to the Indian Ocean and western South Pacific Ocean (**Panel A**), or Pacific Ocean and eastern Indian Ocean (**Panel B**). Results from scenarios with K equal to two through four are shown. Individuals are ordered identically across scenarios for each dataset. Sample collections are shown at bottom of figure and are labeled as in Table 1. Horizontal bars at top correspond with populations resolved in this study.

**Figure S6.** Barplots displaying admixture proportions inferred from STRUCTURE analyses performed without an admixture model of ancestry and using a dataset including all sample collections and 4,165 SNPs. Results from scenarios with K equal to two through seven are shown. Individuals are ordered identically across panels. Sample collections are shown at bottom of figure and are labeled as in Table 1. Horizontal bar at top corresponds with populations resolved in this study.

**Figure S7.** Barplots displaying admixture proportions inferred from STRUCTURE analyses performed without an admixture model of ancestry and using datasets (n = 4,165 SNPs) limited to the Indian Ocean and western South Pacific Ocean (**Panel A**), or Pacific Ocean and eastern Indian Ocean (**Panel B**). Results from scenarios with K equal to two through four are shown. Individuals are ordered identically across scenarios for each dataset. Sample collections are shown at bottom of figure and are labeled as in Table 1. Horizontal bars at top correspond with populations resolved in this study.

**Figure S8.** Per locus F_ST_ values estimated by BayeScan using neutral prior odds of 100:1. Loci putatively under the influence of natural selection were distinguished using a false discovery rate of 0.10.

**Figure S9.** Per locus F_ST_ values estimated using the FDIST2 methodology implemented in Arlequin. A significance threshold of p < 0.05 was used to distinguish loci putatively under the influence of natural selection.

**Figure S10.** Bidirectional relative migration rates among striped marlin (*Kajikia audax*) populations calculated using a dataset where loci not conforming to Hardy-Weinberg equilibrium and selective neutrality were removed (n = 4,106 SNPs). Open circles represent populations, and lines connecting circles are weighted according to relative migration rate. Relative migration rates with 95% confidence intervals larger than 0.00 are denoted with an asterisk. Values shown here were calculated with putative migrants excluded.
